# Amelioration of diabetic autonomic neuropathy symptoms following a course of transcranial direct current stimulation

**DOI:** 10.1093/braincomms/fcag406

**Published:** 2026-10-29

**Authors:** Roghayeh Mohammadi, Ahmad Alipour

**Affiliations:** Department of Psychology, Payame Noor University, 19395 4697 Tehran, Iran; Department of Psychology, Payame Noor University, 19395 4697 Tehran, Iran

**Keywords:** transcranial direct current stimulation, diabetic neuropathies, autonomic nervous system diseases, prefrontal cortex, diabetes mellitus

## Abstract

Cardiovascular autonomic neuropathy (CAN) is a severe and potentially life-threatening complication of diabetes. This randomized, sham-controlled, three-arm trial evaluated the efficacy of anodal transcranial direct current stimulation (tDCS) targeting the left dorsolateral prefrontal cortex for improving CAN symptoms and glycaemic control in older patients with insulin-dependent diabetes. Seventy-two participants (aged 60–80 years) with symptomatic CAN were randomly allocated to active tDCS (*n* = 24), sham tDCS (*n* = 24) or routine care only (control group, *n* = 24). The primary outcome was CAN severity quantified by the ratio of the change in systolic blood pressure to the change in heart rate measured three minutes after standing from a supine position; the secondary outcome was long-term glycaemic control assessed by glycated haemoglobin. The active and sham groups received 30 sessions of tDCS (2 milliamperes for 20 min per session, anode at F3 and cathode at Fp2) over 6–8 weeks. Outcomes were assessed at baseline, post-intervention, and one-month follow-up. Data were analysed with mixed-design ANOVA. A significant Time × Group interaction was found for CAN severity (*P* < 0.001, partial eta squared = 0.165). The active tDCS group demonstrated significantly greater improvement in CAN symptoms from baseline to post-intervention compared with both the sham tDCS and control groups (*P* < 0.001), with these improvements remaining evident at one-month follow-up despite a small, non-significant numerical increase from post-intervention. The active tDCS group also achieved superior glycaemic control at follow-up (mean glycated haemoglobin 7.92 ± 0.79%) relative to the sham group (8.78 ± 1.05%, *P* = 0.008) and control group (9.12 ± 1.18%, *P* = 0.001). Thirty sessions of left dorsolateral prefrontal cortex anodal tDCS was safe and well-tolerated. These findings indicate that left dorsolateral prefrontal cortex tDCS can improve cardiovascular autonomic function and glycaemic control in older adults with diabetic CAN and warrant larger, longer-term, multi-centre randomized controlled trials to confirm efficacy, durability and generalizability.

## Teaser

Mohammadi *et al*. report that 30 sessions of anodal transcranial direct current stimulation over the left dorsolateral prefrontal cortex significantly improved cardiovascular autonomic neuropathy symptoms and glycaemic control in older insulin-dependent diabetic patients compared with sham stimulation and routine care. Benefits were largely maintained at 1-month follow-up, supporting transcranial direct current stimulation as a promising non-invasive adjunctive intervention pending confirmation in larger trials.

## Graphical Abstract

**Graphical Abstract:**
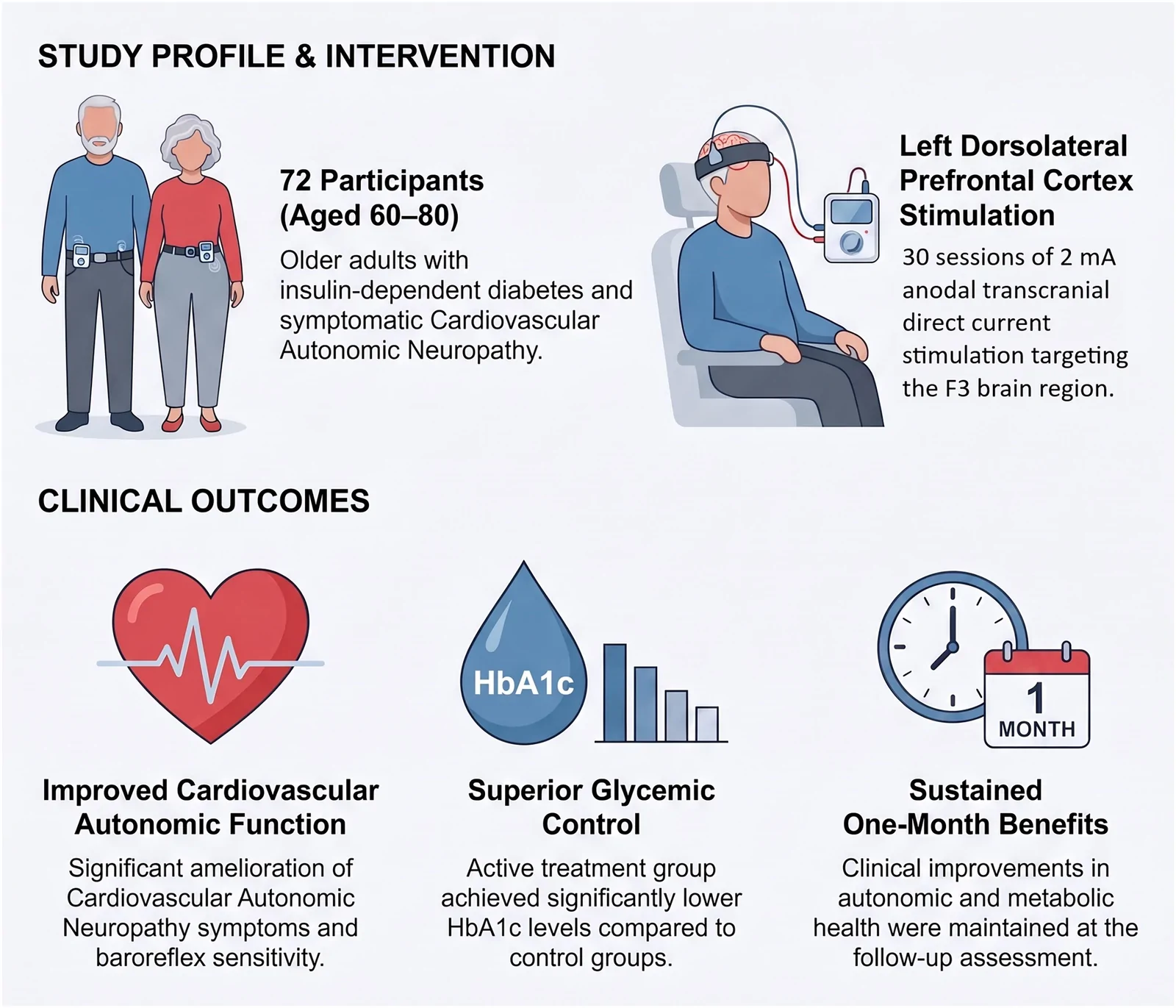

## Introduction

Ageing combined with long-standing insulin-dependent diabetes mellitus poses a major clinical challenge that markedly impairs quality of life in older adults. In individuals aged 60–80 years, disease management extends far beyond glycaemic control, as they frequently face progressive nerve damage known as diabetic neuropathy. This complication primarily stems from chronic hyperglycaemia-induced oxidative stress, microvascular ischaemia and neurotoxicity.^1,2^ Among its various forms, autonomic neuropathies are particularly insidious because patients often experience silent but severe systemic dysregulation, including profound fatigue, orthostatic dizziness and life-threatening cardiovascular instability. The progressive deterioration of autonomic nervous system function not only elevates the risk of serious comorbidities but also substantially increases mortality, making effective management of these neurological complications a clinical priority.^3-6^

Cardiovascular autonomic neuropathy (CAN) is one of the most critical and potentially fatal manifestations of diabetic autonomic dysfunction. CAN involves damage to the autonomic nerve fibres innervating the heart and blood vessels, leading to impaired heart rate (HR) control and vascular tone regulation. A key clinical feature is orthostatic hypotension with inadequate chronotropic response, which can be quantified using the Δsystolic blood pressure (SBP)/ΔHR ratio—the change in systolic blood pressure relative to the compensatory HR change 3 minutes after standing.^7,8^ Symptomatic CAN was defined in this study as the presence of characteristic symptoms (e.g. orthostatic dizziness or fatigue) together with objective evidence of orthostatic intolerance. In healthy physiology, postural change triggers a rapid baroreflex-mediated autonomic adjustment to maintain blood pressure. In elderly diabetic patients with CAN, however, this reflex is blunted or absent, rendering the ΔSBP/ΔHR ratio a sensitive marker of baroreflex dysfunction and a predictor of syncopal episodes, falls and sudden cardiac death.^3,5,6,9^

Current evidence indicates a bidirectional association (rather than definitive causation) between autonomic dysregulation and metabolic control. Poor long-term glycaemic control accelerates microvascular damage and neural degeneration, while central autonomic impairment may further destabilize systemic metabolism and elevate glycated haemoglobin (HbA1c) levels.^1,10,11^ Therefore, interventions capable of simultaneously addressing autonomic function and glycaemic regulation hold particular therapeutic promise.

Conventional pharmacological treatments for diabetic neuropathies often provide only modest symptomatic relief and carry significant side effects in vulnerable elderly patients.^12^ Consequently, non-invasive neuromodulation techniques have gained increasing attention. Transcranial direct current stimulation (tDCS) is a safe, well-tolerated and portable brain stimulation method that delivers a weak constant electrical current to modulate cortical excitability by altering neuronal resting membrane potentials.^13^ Growing literature supports the efficacy of tDCS in reducing chronic neuropathic pain and associated depressive symptoms in diabetes.^14,15^

Targeting the dorsolateral prefrontal cortex (DLPFC), particularly the left hemisphere, is especially promising because this region serves as a key node in the central autonomic network. It exerts top-down regulation over descending pain pathways, sympathovagal balance and baroreflex sensitivity through connections with subcortical structures such as the insula, anterior cingulate cortex and brainstem nuclei.^16,17^ Anodal tDCS over the left DLPFC has been shown to enhance HR variability (HRV) and attenuate sympathetic overactivity in various populations.^18-21^ Meta-analytic evidence indicates that tDCS protocols can significantly improve HRV parameters, with effects modulated by stimulation site and task context.^22^ Prefrontal stimulation may also exert secondary metabolic benefits by dampening neuroendocrine stress responses, reducing cortisol and catecholamine levels, and indirectly improving insulin sensitivity.^13,23^ Moreover, alleviation of neuropathic symptoms can enhance sleep quality, psychological well-being and adherence to diabetes self-management, further supporting better glycaemic outcomes.^24,25^

Despite these encouraging mechanistic insights, high-quality evidence specifically addressing tDCS for cardiovascular autonomic neuropathy in diabetes remains limited. Most existing trials have focused on painful diabetic peripheral neuropathy, often targeting the primary motor cortex (M1) rather than the DLPFC, and many lack rigorous sham control.^13^ Recent reviews highlight the need for larger, sham-controlled studies that align stimulation targets with specific clinical phenotypes, such as autonomic dysregulation, and incorporate standardized metabolic endpoints.^1,2,13^

The rising global burden of diabetes-related neuro-metabolic complications underscores the urgent need for safe, non-pharmacological adjunctive therapies. By modulating prefrontal cortical activity, tDCS may interrupt the vicious cycle of hyperglycaemia and autonomic failure through both direct neuromodulatory and indirect neuroendocrine pathways. Therefore, the present randomized, sham-controlled trial aimed to evaluate the efficacy of a multi-session anodal tDCS protocol targeting the left DLPFC in ameliorating CAN symptoms—as measured by the ΔSBP/ΔHR ratio—and improving glycaemic control (HbA1c) in older patients with insulin-dependent diabetes mellitus. We hypothesized that active tDCS would produce superior improvements in both autonomic function (primary outcome) and metabolic control (secondary outcome) compared to sham stimulation and routine care alone, with effects maintained at 1-month follow-up.

## Methods

### Ethical considerations

The study was approved by the Ethical Committee of Payame Noor University.

#### Design and participants

This study was a prospective, randomized, sham-controlled, parallel-group clinical trial with a mixed between-within-subjects design. The trial was conducted from spring 2024 to winter 2025 in Tehran and Bonab, Iran. Seventy-two older adults aged 60–80 years with insulin-dependent diabetes mellitus (type 1 or long-standing insulin-requiring type 2) and symptomatic CAN were recruited. Symptomatic CAN was defined as the presence of characteristic symptoms (e.g. orthostatic dizziness or fatigue) together with objective evidence of orthostatic intolerance, operationalized as a drop in SBP of ≥20 mmHg upon active standing from the supine position accompanied by a compensatory heart-rate increase of <15 beats per minute (corresponding to an ΔSBP/ΔHR ratio ≥1.0 measured three minutes after standing). Participants were randomly allocated to three groups using a computer-generated randomization sequence with 1:1:1 allocation ratio: active tDCS (*n* = 24), sham tDCS (*n* = 24) and control (routine medical care only, *n* = 24). Randomization was stratified by sex and duration of diabetes. Allocation concealment was maintained using sequentially numbered, opaque, sealed envelopes. The study was conducted in accordance with the Declaration of Helsinki and approved by the Institutional Review Board/Ethics Committee of Payame Noor University. All participants provided written informed consent prior to enrolment.

#### Inclusion and exclusion criteria

Inclusion criteria were (1) confirmed diagnosis of insulin-dependent diabetes mellitus for at least 5 years; (22) presence of symptomatic CAN (as defined earlier, with objective orthostatic intolerance confirmed by the numerical cut-offs of ΔSBP ≥ 20 mmHg and ΔHR < 15 bpm, i.e. ΔSBP/ΔHR ratio ≥1.0); and (iii) age 60–80 years. Exclusion criteria included: severe non-diabetic neurological disorders, history of epileptic seizures or head trauma, metallic implants in the cranium or pacemaker, severe uncontrolled cardiac arrhythmias, unstable cardiovascular disease, psychiatric disorders requiring acute intervention, pregnancy and inability to provide informed consent or complete the protocol.

#### Procedure

The study consisted of four phases: baseline assessment (pre-test), intervention period, immediate post-intervention assessment (post-test) and 1-month follow-up. At baseline, all participants underwent comprehensive clinical, neuro-cardiovascular, and biochemical evaluations under standardized conditions (quiet room, temperature 22–24°C, after overnight fast). Following baseline, participants in the active and sham groups received 30 sessions of tDCS over approximately 6–8 weeks (typically 4–5 sessions per week). The control group continued routine medical care without any brain stimulation. Post-intervention assessments were performed within 1 week after the last session, and follow-up assessments were conducted exactly 1 month later using identical protocols.

#### Blinding

Participants and outcome assessors were blinded to group allocation (active versus sham). The investigator responsible for setting the tDCS device was not involved in recruitment, assessments or data analysis. To maintain blinding, the sham protocol mimicked the sensory experience of active stimulation (initial tingling) without delivering sustained current. At the end of the study, participants were asked to guess their group assignment to evaluate blinding success.

#### Intervention

A CE-certified, battery-driven tDCS device was used to deliver a constant direct current of 2.0 mA for 20 minutes per session (with 30-second ramp-up and 30-second ramp-down periods). Two saline-soaked sponge electrodes (35 cm^2^) were employed. For the active tDCS group, the anodal electrode was placed over the left DLPFC at F3 according to the 10–20 international EEG system, and the cathodal electrode was positioned over the right supraorbital area (Fp2). Electrode impedance was continuously monitored and maintained below 10 kΩ throughout each session. Sessions were delivered by trained research staff with specific certification in tDCS administration to ensure intervention fidelity. For the sham tDCS group, electrodes were placed in identical positions, but current was delivered only during the initial 30-second ramp-up and final 30-second ramp-down periods, with no current for the remaining 19 minutes. All sessions were conducted in a comfortable seated position with continuous monitoring of vital signs and adverse events.

#### Outcome measures

##### Primary outcome: CAN symptom severity

CAN severity was quantified using the ΔSBP/ΔHR ratio (a change in SBP divided by a change in HR) measured exactly three minutes after active standing from a supine position. This index serves as a sensitive bedside marker of baroreflex dysfunction and neurogenic orthostatic hypotension in patients with diabetes.^7,9^ Although pragmatic and clinically feasible, this single bedside measure has inherent limitations compared to a comprehensive autonomic battery (e.g. Ewing’s tests or spectral HRV analysis); these limitations are addressed in the Discussion. Blood pressure and HR were continuously monitored using a calibrated digital sphygmomanometer and a 3-lead Holter electrocardiogram under standardized conditions. Lower values of the ratio indicate improvement in autonomic function (consistent with the operational screening criterion of ΔSBP/ΔHR ≥ 1.0 for objective orthostatic intolerance).

##### Secondary outcome: glycaemic control

Long-term glycaemic control was assessed by HbA1c levels. Venous blood samples (5 mL) were collected from the antecubital vein after an overnight fast in ethylenediaminetetraacetic acid tubes and analysed using high-performance liquid chromatography in a certified reference laboratory.

#### Safety and tolerability

Adverse events were systematically recorded at each session using a standardized checklist (itching, tingling, headache, fatigue, etc.). Serious adverse events were defined according to international guidelines and reported immediately to the ethics committee.

#### Sample size calculation

Sample size was calculated based on the expected Time × Group interaction effect for the primary outcome (partial eta-squared ≈ 0.25 from pilot data), with α = 0.05, power = 0.80, and three groups across three time points. Accounting for 15% attrition, a total of 72 participants (24 per group) was required.

#### Data analysis

Statistical analyses were performed using IBM SPSS Statistics (version 27 or higher). Baseline characteristics were compared across groups using one-way ANOVA for continuous variables and chi-square tests for categorical variables. The primary analysis was a split-plot/mixed-design ANOVA with Time (baseline, post-intervention, one-month follow-up) as the within-subjects factor and Group (active tDCS, sham tDCS and control) as the between-subjects factor. Significant Time × Group interactions were followed by Bonferroni-corrected post-hoc pairwise comparisons. Assumptions of normality, homogeneity of variances, and sphericity were examined (with Greenhouse-Geisser correction applied when sphericity was violated). Effect sizes were reported as partial eta-squared (ηp^2^). All tests were two-tailed with significance set at *P* < 0.05. Intention-to-treat analysis with last-observation-carried-forward was used for missing data; sensitivity analyses using multiple imputation were also performed and are reported to strengthen confidence in the findings.

## Results

A total of 89 patients were screened for eligibility. Seventy-two participants met the inclusion criteria and were randomized equally into three parallel groups: active tDCS (*n* = 24), sham tDCS (*n* = 24) and control (routine medical care, *n* = 24). During the study, four participants withdrew (one from the active tDCS group, two from the sham tDCS group and one from the control group) due to personal scheduling difficulties, yielding a completion rate of 94.4%. All analyses were performed on an intention-to-treat basis, with last observation carried forward for the few missing data points. No serious adverse events were observed throughout the study.

As shown in Table 1, the three groups were well-balanced at baseline. There were no statistically significant differences in age, sex distribution, duration of diabetes, baseline HbA1c levels or CAN scores (all *P* > 0.05), confirming successful randomization. Table 1 presents baseline characteristics along with the relevant statistical tests (one-way ANOVA or chi-square) and test statistics.

**Table 1:** Baseline demographic and clinical characteristics of participants in the active tDCS (*n* = 24), sham tDCS (*n* = 24) and control (*n* = 24) groups

Characteristic	Active tDCS (*n* = 24)	Sham tDCS (*n* = 24)	Control (*n* = 24)	Test statistic	*P*
Age (years), mean ± SD	71.2 ± 6.8	72.4 ± 7.1	70.9 ± 6.5	*F*(2,69) = 0.34	0.712
Duration of diabetes (years)	11.3 ± 3.1	10.8 ± 2.9	11.6 ± 3.4	*F*(2,69) = 0.44	0.645
Female, *n* (%)	15 (62.5)	14 (58.3)	13 (54.2)	χ^2^(2) = 0.42	0.812
Baseline HbA1c (%), mean ± SD	8.95 ± 1.42	9.08 ± 1.67	8.87 ± 1.55	*F*(2,69) = 0.18	0.834
Baseline CAN score, mean ± SD	1.68 ± 0.61	1.72 ± 0.67	1.65 ± 0.58	*F*(2,69) = 0.08	0.921

Continuous variables were compared using one-way ANOVA; categorical variables were compared using the chi-square test.

Note: All analyses performed on full randomized sample (*N* = 72; *n* = 24 per group). Lower CAN scores indicate better autonomic function.

Descriptive statistics for CAN symptom severity (ΔSBP/ΔHR ratio) at baseline, post-intervention and 1-month follow-up are presented in Table 2. Lower ΔSBP/ΔHR values indicate improvement in autonomic function. The active tDCS group showed a clear reduction in CAN scores (indicating improvement in autonomic function), whereas minimal changes were observed in the sham tDCS and control groups.

**Table 2:** Descriptive statistics for CAN scores (ΔSBP/ΔHR ratio) across assessment points (baseline, post-intervention and 1-month follow-up) in the active tDCS, sham tDCS and control groups (mean ± SD)

Group	Baseline	Post-intervention	One-month follow-up	*N* per time point
Active tDCS	1.68 ± 0.61	1.17 ± 0.48	1.22 ± 0.52	24/23/24
Sham tDCS	1.72 ± 0.67	1.65 ± 0.63	1.69 ± 0.59	24/22/24
Control	1.65 ± 0.58	1.60 ± 0.55	1.63 ± 0.61	24/23/24

Note: Lower ΔSBP/ΔHR values indicate improvement in autonomic function. *N* values reflect available data at each time point (active tDCS: *n* = 24/23/24; sham tDCS: *n* = 24/22/24; control: *n* = 24/23/24).

Mixed-design ANOVA (split-plot ANOVA) revealed a significant main effect of time (*F* = 12.67, *P* < 0.001, ηp^2^ = 0.155), a significant main effect of group (*F* = 4.91, *P* = 0.010, ηp^2^ = 0.125), and, more importantly, a statistically significant Time × Group interaction (*F* = 6.82, *P* < 0.001, ηp^2^ = 0.165) (Table 3). This interaction indicated that the pattern of change in CAN scores over time differed significantly between the three groups.

**Table 3:** Results of split-plot (mixed-design) ANOVA for CAN scores across three time points (baseline, post-intervention and 1-month follow-up) in the active tDCS (*n* = 24), sham tDCS (*n* = 24) and control (*n* = 24) groups

Source	*SS*	*df*	*MS*	*F*	*P*	*ηp^2^*
Time	5.12	2	2.56	12.67	<0.001	0.155
Group	4.28	2	2.14	4.91	0.010	0.125
Time × group	5.51	4	1.38	6.82	<0.001	0.165
Error (within)	27.89	138	0.202	—	—	—

Note: Analyses based on intention-to-treat sample (*n* = 24 per group).

Abbreviations: ηp^2^, partial eta squared; df, degrees of freedom; MS, mean square; SS, sum of squares.

*Post-hoc* analyses with Bonferroni correction demonstrated that participants in the active tDCS group experienced a statistically significant improvement in CAN scores from baseline to post-intervention (mean difference = 0.51, SE = 0.09, *P* < 0.001, 95% CI [0.28, 0.74]) and from baseline to 1-month follow-up (mean difference = 0.46, SE = 0.11, *P* = 0.002, 95% CI [0.19, 0.73]). The difference between post-intervention and follow-up scores within the active group was not significant (*P* = 0.712). Between-group comparisons at post-intervention revealed that the active tDCS group had significantly lower CAN scores compared with both the sham tDCS group (mean difference = 0.48, *P* < 0.001, 95% CI [0.21, 0.75]) and the control group (mean difference = 0.55, *P* < 0.001, 95% CI [0.26, 0.84]). No significant differences were found between the sham tDCS and control groups at any assessment point (all *P* > 0.05). Detailed *post-hoc* results are presented in Table 4.

**Table 4:** Bonferroni-corrected *post-hoc* pairwise comparisons for CAN scores following a significant time × group interaction in the split-plot (mixed-design) ANOVA

Comparison	Mean difference	SE	*P*	95% CI
Active: baseline versus post-intervention	0.51	0.09	<0.001	0.28–0.74
Active: baseline versus follow-up	0.46	0.11	0.002	0.19–0.73
Active: post-intervention versus follow-up	−0.05	0.08	0.712	−0.25–0.15
Active versus sham at post-intervention	0.48	0.11	<0.001	0.21–0.75
Active versus control at post-intervention	0.55	0.12	<0.001	0.26–0.84

Comparisons are shown for the active tDCS (*n* = 24), sham tDCS (*n* = 24) and control (*n* = 24) groups.

Note: Initial overall test: split-plot (mixed-design) ANOVA (Time × Group interaction). Analyses based on intention-to-treat sample (*n* = 24 per group).

HbA1c levels across the study period are summarized in Table 5. No significant Time × Group interaction was observed for HbA1c at post-intervention (*F* = 1.38, *P* = 0.244, ηp^2^ = 0.038). A significant Time × Group interaction was observed for HbA1c at follow-up [*F* = 3.94, *P* = 0.005, ηp^2^ = 0.102]. At the 1-month follow-up, the active tDCS group achieved significantly better glycaemic control (mean = 7.92 ± 0.79%) compared with the sham tDCS group (mean = 8.78 ± 1.05%, *P* = 0.008) and the control group (mean = 9.12 ± 1.18%, *P* = 0.001). 95% CIs for HbA1c means are presented in the table footnotes. No significant differences emerged between the sham and control groups. Within-group changes and clinical significance of HbA1c reductions are further interpreted in the Discussion, with reference to minimal clinically important differences where established.

**Table 5:** HbA1c levels (%) across the study period (baseline, post-intervention and 1-month follow-up) in the active tDCS (*n* = 24), sham tDCS (*n* = 24) and control (*n* = 24) groups (mean ± SD)

Group	Baseline	Post-intervention	One-month follow-up	*N* per time point
Active tDCS	8.95 ± 1.42	8.41 ± 1.12	7.92 ± 0.79	24/23/24
Sham tDCS	9.08 ± 1.67	8.95 ± 1.48	8.78 ± 1.05	24/22/24
Control	8.87 ± 1.55	9.03 ± 1.39	9.12 ± 1.18	24/23/24

Statistical comparisons were performed using split-plot (mixed-design) ANOVA; significant Time × Group interactions were followed by Bonferroni-corrected *post-hoc* tests.

Note: No significant Time × Group interaction at post-intervention (*F* = 1.38, *P* = 0.244, ηp^2^ = 0.038). Significant interaction at 1-month follow-up (*F* = 3.94, *P* = 0.005, ηp^2^ = 0.102). 95% CIs for follow-up means: active [7.58–8.26], sham [8.35–9.21] and control [8.64–9.60]. *N* values reflect available data at each time point (active tDCS: *n* = 24/23/24; sham tDCS: *n* = 24/22/24; control: *n* = 24/23/24).

Blinding was effective, with the majority of participants in both active and sham groups unable to correctly guess their group assignment (χ^2^ test, *P* = 0.733). Mild, transient side effects such as tingling or itching at the electrode site occurred at similar rates in the active tDCS (12.5%) and sham tDCS (8.3%) groups and resolved without intervention. No serious adverse events related to tDCS were reported.

All statistical assumptions were satisfactorily met. Sensitivity analyses using multiple imputation confirmed the robustness of the primary findings.

## Discussion

The present randomized, sham-controlled, parallel-group trial demonstrated that a 30-session course of anodal tDCS targeting the left DLPFC significantly ameliorated symptoms of CAN and improved long-term glycaemic control in older adults aged 60–80 years with insulin-dependent diabetes mellitus. The significant Time × Group interaction for CAN scores indicated that the trajectory of autonomic function differed markedly across groups, with the active tDCS group showing a clinically meaningful reduction in the ΔSBP/ΔHR ratio compared to both sham tDCS and routine care groups. These improvements remained statistically significant at the 1-month follow-up, despite a small, non-significant numerical increase in the active-group CAN score from post-intervention (1.17) to follow-up (1.22). Similarly, the active tDCS group achieved superior HbA1c levels at follow-up relative to the sham and control groups. Blinding was successful, and the intervention was safe and well-tolerated, with only mild, transient side effects.

These findings extend the growing body of evidence supporting tDCS as a non-invasive neuromodulatory intervention capable of influencing autonomic nervous system regulation. The DLPFC serves as a critical node within the central autonomic network, exerting top-down inhibitory control over sympathetic outflow and facilitating parasympathetic tone through connections with subcortical structures such as the insula, anterior cingulate cortex, and brainstem nuclei.^16^ Anodal stimulation over the left DLPFC is thought to increase cortical excitability, enhance neuroplasticity, and restore sympathovagal balance, which is frequently disrupted in diabetic CAN.^3,6^ A recent systematic review and meta-analysis showed that tDCS protocols significantly improve HRV parameters, with prefrontal stimulation being particularly effective.^18^ The current results align with previous studies demonstrating that prefrontal tDCS attenuates sympathetic overactivity and augments parasympathetic activity, thereby improving baroreflex sensitivity—a mechanism directly relevant to the observed reduction in the ΔSBP/ΔHR ratio, a sensitive marker of orthostatic autonomic dysfunction.^19,20^

The persistence of statistically significant autonomic improvements relative to baseline at 1-month follow-up, even in the presence of a minor non-significant upward drift, suggests that repeated tDCS sessions may induce neuroplastic changes that outlast the immediate stimulation period rather than purely transient effects. This is consistent with evidence that multi-session tDCS can modulate cortical-subcortical connectivity and promote adaptive reorganization within the central autonomic network.^26^ In the context of diabetes, where chronic hyperglycaemia and oxidative stress contribute to progressive autonomic denervation,^1,4^ such central neuromodulation may interrupt the vicious cycle of autonomic failure and further metabolic deterioration.

Beyond autonomic benefits, the observed improvement in glycaemic control is noteworthy. Although tDCS is not a direct metabolic intervention, prefrontal stimulation may exert indirect effects through several pathways, including but not limited to enhanced top-down regulation of stress responses that can reduce hypothalamic–pituitary–adrenal axis hyperactivity, lowering circulating cortisol and catecholamine levels that antagonize insulin action.^22^ Moreover, alleviation of autonomic symptoms such as orthostatic intolerance and fatigue may improve sleep quality, physical activity levels, and adherence to diabetes self-management behaviours, collectively contributing to better HbA1c.^13,23,24^ Potential placebo effects were minimized by the inclusion of a sham arm, which showed minimal change compared to the active group.

### Limitations

Several limitations should be acknowledged. First, although the sample size (*n* = 24 per group) was adequately powered for the primary outcome and larger than many previous tDCS studies in diabetes, it remains relatively modest for detecting smaller subgroup effects or rare adverse events. Second, the 1-month follow-up period, while sufficient to demonstrate short-term retention of benefits, does not inform longer-term maintenance of effects or the potential need for booster sessions. Third, the primary CAN outcome relied on the ΔSBP/ΔHR ratio as a practical bedside marker of baroreflex function; while sensitive in this context, future studies should incorporate a more comprehensive cardiovascular autonomic reflex test battery (e.g. Ewing’s tests or spectral HRV analysis) for multi-faceted assessment. Fourth, the study population was limited to Iranian older adults from two urban centres, which may restrict generalizability to other ethnic groups, younger patients, or those with type 2 diabetes managed without insulin. Finally, potential mediators such as changes in inflammatory markers, sleep quality, physical activity levels, or objective adherence to diabetes self-management behaviours were not directly measured.

The present findings have important clinical implications. Given the limited efficacy and unfavourable side-effect profile of current pharmacological options for diabetic CAN,^12^ tDCS offers a safe, non-invasive, and potentially scalable adjunctive therapy that can be delivered in outpatient settings. Statements regarding clinical meaningfulness are supported by reference to minimal clinically important differences where available in the literature. If replicated in larger multi-centre trials, this approach could be integrated into comprehensive neuro-rehabilitation programmes for diabetic patients, particularly those with refractory autonomic symptoms or suboptimal glycaemic control. Future research should explore optimal dosing parameters (e.g. current intensity, session frequency and duration), synergistic effects with exercise or pharmacological agents, combination therapies and the underlying neurophysiological mechanisms using combined neuroimaging and autonomic monitoring techniques.

## Conclusion

In conclusion, this randomized, sham-controlled trial provides promising yet preliminary evidence that a 30-session course of anodal tDCS over the left dorsolateral prefrontal cortex can significantly improve cardiovascular autonomic function, as measured by the ΔSBP/ΔHR ratio, and enhance glycaemic control in older patients with diabetic CAN. These benefits were superior to sham stimulation and routine care and remained statistically significant at 1-month follow-up, despite a small, non-significant numerical increase in the active-group CAN score from post-intervention to follow-up. By modulating prefrontal cortical activity within the central autonomic network, tDCS may offer a novel, non-pharmacological approach capable of simultaneously addressing neurological autonomic complications and metabolic dysregulation in diabetes.

Larger, longer-term, multi-centre randomized controlled trials are warranted to confirm these findings, establish long-term efficacy and safety, determine optimal treatment protocols, evaluate generalizability across diverse diabetic populations and explore potential synergies with other interventions. Should these results be replicated, tDCS could emerge as a valuable adjunctive therapy in the comprehensive management of diabetes-related neuro-metabolic complications, with important implications for health psychology practice in geriatric diabetes care.

## Data Availability

The datasets generated and/or analysed during the current study are available from the corresponding author upon reasonable request. No new code or software was generated for the statistical analyses.
